# Multicolor imaging in choroidal osteomas

**DOI:** 10.1186/s40942-018-0150-y

**Published:** 2018-12-13

**Authors:** Ramesh Venkatesh, Bharathi Bavaharan, Naresh Kumar Yadav, Kumar Saurabh, Priya Srinivasan, Padmalini Mahendradas, Vishma Prabhu, Rupak Roy

**Affiliations:** 1 Department of Retina and Vitreous, Narayana Nethralaya, #121/C, 1st R Block, Chord Road, Rajaji Nagar, Bengaluru, Karnataka 560010 India; 2 Department of Retina and Vitreous, Aditya Birla Sankara Nethralaya, No. 147, Mukundapur EM By Pass, Near Purva Jadavpur Thana, Kolkata, West Bengal 700099 India

**Keywords:** Choroidal osteoma, Multicolour, Imaging, Optical coherence tomography, Fundus autofluoroscence

## Abstract

**Background:**

To describe the multicolour (MC) imaging characteristics associated with choroidal osteomas (CO) and their secondary complications.

**Methods:**

Retrospective descriptive case series of eleven eyes of ten patients with CO. Findings of multicolour imaging were correlated with visual acuity, clinical features, lesion characteristics and findings from other imaging modalities.

**Results:**

Infrared reflectance (IR) images showed calcified CO lesions as hyporeflectance (dark) areas while decalcified lesions were seen as iso reflectance (normal) areas. Overlying RPE atrophy on IR were seen as white areas. MC images showed color variations depending upon the reflectivity of the tumour material tumour and retinal pigment epithelial (RPE) atrophy. Green color was noted in calcified CO tumour while decalcified CO tumour showed no color change. RPE atrophy were seen as bright orange areas. Green and blue reflectance images were not able to pick the choroidal osteoma lesion. Other changes secondary to CO like presence of choroidal neovascular membrane, hemorrhage and/or fluid in the retinal layers were identified on green and blue reflectance images.

**Conclusion:**

MC imaging is a useful tool in our arsenal of existing imaging modalities in the assessment of CO and its secondary changes. Change in reflectance of the IR and MC images can be used as an indicator to assess the extent of tumour decalcification and its secondary changes and therefore, can aid in prognostication in the same. It has the potential to replace color fundus photography in documentation and follow up of patients with CO.

## Background

Choroidal osteoma (CO) was first described by Gass et al. [1] in 1978 as a rare benign ossifying tumour of the choroid. It is typically unilateral in 80% of cases and is seen in young healthy females. It arises in late childhood or early adulthood and its most common symptoms are blurred vision, metamorphopsia and presence of a scotoma [2]. The CO tends to be located in the juxta or peri papillary area and may extend into the macular region. The clinical appearance of the tumour may vary from well-defined, white-cream or yellow-grey to orange lesion which corresponds to the grade of ossification (orange pigmentation is present in areas with more ossification) [3]. Varying degrees of clumping of orange, yellow and brown pigment can be identified on the tumour surface. Multiple retinal imaging modalities have been used for studying CO and its secondary ocular findings. Over time, ocular ultrasound (US), fluorescein angiography (FA) and optical coherence tomography (OCT) have been widely used for diagnosis and follow-up of eyes with CO. Enhanced-depth imaging OCT (EDI-OCT) is a recent addition of OCT, that has been able to reveal the presence of bone lamella, tubular lamella with optically empty centre, vascular channels and trabecular bone in patients with CO [4, 5]. Multicolor (MC) scanning laser imaging is a recently introduced innovative technology developed for Spectralis SD-OCT (Heidelberg Engineering, Heidelberg, Germany). It has been widely used to describe the findings in various retinal pathologies [6]. MC imaging is a novel, non-invasive retinal imaging modality that simultaneously acquires three reflectance images of the retina using light of 3 different wavelengths and then producing a composite image, thereby allowing analysis of changes at various levels within the retina [7]. MC imaging findings in eyes with CO have not yet been described.

The aim of the study is to describe the MC imaging features in CO in conjunction with color fundus photography (CFP), fundus autofluoroscence (FAF) and OCT.

## Methods

In this retrospective study, CO cases who had undergone multimodal fundus imaging were collected from 2 tertiary retina referral centres (Narayana Nethralaya, Bangalore and Sankara Nethralaya, Kolkata). The diagnosis was based on the presence of a yellow-white to orange-red mass deep to the retinal pigment epithelium (RPE) and bone density on ultrasonography. All patients had undergone CFP, FAF, OCT and MC imaging in the study. Institutional review board approval was obtained for this retrospective study.

The clinical details of each patient were collected retrospectively by review of records, including age, gender, laterality and ocular symptoms. Each patient underwent complete ocular examination including best-corrected visual acuity, slit-lamp examination, and indirect ophthalmoscopy. Lesion characteristics recorded for each eye included lesion location within the fundus, extent of calcification or decalcification, and the presence or absence of sub retinal fluid (SRF) or intraretinal fluid (IRF) with/without choroidal neovascular membrane (CNV).

Color fundus photography was done using either Optos Daytona ultra-wide field color fundus camera (Optos Daytona; Optos PLC, United Kingdom) or Zeiss 450 Fundus camera. Data included foveal involvement by the tumour (foveal, extrafoveal), tumour color (yellow, orange, white) and presence of calcification or decalcification. Calcification was defined as an orange lesion in the fundus located deep underneath the intact RPE and no visible choroidal vessels. Decalcification (complete, partial) was defined as pale areas within the osteoma, with overlying RPE thinning and visibility of underlying choroidal vessels.

All patients had OCT, FAF and MC imaging performed by confocal scanning laser ophthalmoscope using a Heidelberg Spectralis HRA-OCT (Heidelberg Engineering, Dossenheim, Germany). OCT data included tumour surface configuration (flat or depressed), effects of tumour on overlying retina (RPE, photoreceptor and inner retina status), presence of SRF/IRF with/without CNV. Tumour depth was measured using the calliper function through the epicentre of the tumour. The findings of FAF were described as iso, hypo or hyper AF. The patterns described on MC imaging with infrared reflectance (IR), green reflectance (GR) and blue reflectance (BR) were described as dark areas (hypo) or white areas (hyper) or no changes. On MC images, the color of the lesion varied from greenish to yellowish orange to orange depending upon the state of tumour calcification and RPE atrophy. The scanning field was 30° in most cases. 55° field images were obtained in eyes with large sized lesions. In addition, FA, ICGA and OCT-A were performed in select patients only when a suspicion of CNV was present.

We analysed the MC imaging features in CO and corelated it with that of CFP, FAF and OCT using the HRA-OCT unit.

## Results

There were 11 eyes of 10 Asian patients included in the study. The clinical and demographic features are mentioned in Table 1. There were equal number of males and females in the study (5 each). The mean age of patients was 30.63 years (range 16–44 years). Right eye and left eye were involved in 5 and 6 eyes respectively. One patient had bilateral CO while the remaining 9 patients had unilateral CO. Initial symptoms included blurred vision in 7 patients, metamorphopsia in 1 patient and 2 patients were asymptomatic. Visual acuity was between 20/20 and 20/200 in 8 eyes and < 20/200 in 3 eyes. Poor visual acuity (20/200 or worse) was related to foveal photoreceptor layer loss overlying the decalcified tumour in one eye and subfoveal CNV in 2 eyes. On examination, the tumour looked completely calcified in 4 eyes, partly decalcified in 4 eyes and totally decalcified in 3 eyes. Features of CO on CFP, OCT, FAF and MC imaging are described in detail in Table 2. On CFP, the color of the tumour varied from orange (n = 3) to yellowish orange (n = 5) to greyish white (n = 3) depending upon the extent of ossification (orange indicating complete ossification, yellowish orange indicating incomplete decalcification and greyish white indicating incomplete decalcification). The tumour involved the fovea in 9 eyes and was extrafoveal in location in 2 eyes only.

**Table 1:** Demographic and clinical findings in patients with choroidal osteoma

Patient	Involved eye	Age	Symptoms	VA	Clinical examination				Treatment
					Tumour color	Foveal involvement	Type	Associated ocular findings	
1	RE	23/M	None	20/20	Orange	Yes	C	MNF	Observation
2	LE	22/M	BOV	20/400	Orange	Yes	C	FCE	Observation
3	LE	16/M	BOV	20/30	Yellow	Yes	DC	Nil	Observation
4	RE	41/F	BOV	20/20	Yellowish orange	Yes	C	Nil	Observation
	LE	41/F	BOV	20/400	Greyish white	Yes	DC	FCE, CNV	IV Anti-VEGF
5	RE	40/M	Metamorphosia	20/80	Greyish white	Yes	DC	FCE, CNV	IV Anti-VEGF
6	RE	37/F	BOV	20/60	Yellowish orange with white areas in the macula	Yes	PD	CNV	IV Anti-VEGF
7	LE	36/M	None	20/20	Orange	No	C	Nil	Observation
8	LE	18/F	BOV	< 20/400	Yellowish orange with fresh SRH	No	PD	CNV	IV Anti-VEGF
9	RE	19/F	BOV	20/60	Greyish-white with yellowish pigmentation noted at the macula	Yes	PD	FCE	Observation
10	LE	44/F	BOV	20/30	Yellowish orange	Yes	PD	Scarred CNV	Observation

*VA* visual acuity, *RE* right eye, *LE* left eye, *M* male, *F* female, *BOV* blurring of vision, *SRH* sub retinal hemorrhage, *C* calcified, *PD* partly decalcified, *DC* decalcified, *MNF* myelinated nerve fibre, *FCE* focal choroidal excavation, *CNV* choroidal neovascular membrane, *VEGF* vascular endothelial growth factor

**Table 2:** Correlation of color fundus, OCT, fundus autofluoroscence and multi color imaging findings in patients with choroidal osteoma

No.	CFP findings			OCT findings					FAF findings	MC imaging findings			
	Tumour color	Type	Other ocular findings	CRT	Tumour depth	Tumour texture	RPE status	SRF/IRF		MC	IR	GR	BR
1	Orange	C	MNF	198	549	Homogenous	Intact	No	ISO AF	Green	Dark areas	No changes visible	No changes visible
2	Orange	C	FCE	215	956	Homogenous	Intact	No	Predominantly ISO AF	Green	Dark areas	No changes visible	No changes visible
3	Yellow white	DC	Nil	281	907	Homogenous	Intact	SRF+	ISO AF	No color change seen in the tumour region. Orange areas are seen temporally	No change is noted in the region of tumour. White areas corresponding to the RPE atrophy is noted temporal to the macula	No changes visible	No changes visible
4	Yellow orange	C	Nil	241	754	Heterogenous	Intact	No	ISO AF	Green areas with orangish pigmentation noted at the fovea	Dark with white areas with bright white spots at the fovea	No changes visible	No changes visible
	Grey white	DC	FCE, CNV	303	407	Heterogenous	Disrupted	SRF+ IRF+	Predominantly hyper AF with areas of hypo AF	Yellowish green with focal bright orange area at the macula	Mixture of dark and white areas with bright white area at the foveal region	White areas noted nasal to the macula	Minimally white areas
5	Grey white	DC	FCE, CNV	305	Could not be measured	Homogenous	Disrupted	SRF+ IRF+	Predominantly hypo AF	Predominantly bright orange areas	Predominantly White areas	Minimally white areas	Minimally white areas
6	Grey white	PD	CNV	297	579	Heterogenous	Disrupted	IRF+	Predominantly hypo AF with areas of stippled hyper AF	Green areas are seen superior to the optic disc and macula. White areas at the posterior pole are noted	Dark areas are seen superior to the optic disc and macula. White areas corresponding to the CNV is noted at the posterior pole	Bright white area corresponding to the CNV is noted at the posterior pole. Dark area seen at the macula is suggestive of hemorrhage	Minimally white area corresponding to the CNV is noted at the posterior pole. Dark area seen at the macula is suggestive of hemorrhage
7	Orange	C	Nil	146	482	Homogenous	Intact	No	Iso AF	Green	Dark areas	No changes visible	No changes visible
8	Yellow orange with fresh SRH	PD	CNV	328	545	Heterogenous	Disrupted	SRF+	Predominantly iso-AF noted at the tumour region. Minimally Hypo AF inferior to arcade. Hypo AF at the macula s/o fresh SRH with hyper AF above it s/o altered SRH	Green colour noted at the tumour region. Dark red area at the macula s/o SRH. Bright orangish area superiorly s/o RPE atrophy	Dark areas noted at the tumour region. Dark areas at the macula s/o SRH. White areas noted superior to the SRH s/o RPE atrophy	Dark areas at the macula s/o SRH	No changes visible
9	Orange with yellow pigmentat the macula	C	FCE	241	498	Heterogenous	Intact	SRF+	Patches of stippled hyper AF with hyper AF spots at the macula	No color change noted in the tumour area. Yellow spots noted at the macula	No visible color changes are noted in the tumour region. Bright white spots noted in the foveal region	Bright white spots noted at fovea	Bright white spots noted at fovea
10	Yellow orange	PD	Scarred CNV	304	576	Heterogenous	Disrupted	IRF+	Predominantly hypo AF with areas of stippled hyper AF	Predominantly green areas in the region of tumour with bright orange areas s/o RPE atrophy	Dark areas along the superior arcade s/o calcified tumour. Areas of normal reflectance is noted at the posterior pole s/o decalcified lesions. Bright white spots are noted in the macula suggestive RPE atrophy	Mixture of dark and white areas	Mixture of dark and white areas

*CFP* color fundus photography, *OCT* optical coherence tomography, *FAF* fundus autofluoroscence, *MC* multi color, *CRT* central retinal thickness, *RPE* retinal pigment epithelium, *SRF* subretinal fluid, *IRF* intraretinal fluid, *IR* infrared reflectance, *GR* green reflectance, *BR* blue reflectance, *AF* auto fluorescence, *SRH* sub retinal hemorrhage, *C* calcified, *PD* partially decalcified, *DC* decalcified, *MNF* myelinated nerve fibre, *FCE* focal choroidal excavation, *CNV* choroidal neovascular membrane

The OCT data was recorded by evaluating the scans passing through the macula and over the CO lesion. The mean central retinal thickness was 259.91 µm (range 146–328 µm). The mean tumour thickness was 625.3 µm (range 407–956 µm). The retinal pigment epithelium (RPE) overlying the tumour was intact in 6 eyes while it was disrupted/irregular in remaining 5 eyes. In addition, CNV formation was noted in 5 eyes, focal choroidal excavation was seen in 4 eyes while SRF without CNV was seen in 2 eyes.

Fundus autofluorescence findings varied depending on the ophthalmoscopic appearance of each lesion. Heterogeneous lesions exhibited multiple FAF patterns that depended on the characteristics of those corresponding portions of the lesions. Of the 11 eyes with lesions, 5 eyes had areas of iso AF while the remaining 6 eyes had a combination of hyper and hypo AF areas. The findings on FAF depended on the tumour characteristics like the extent of calcification and also on associated retinal findings like presence of CNV, hemorrhage, SRF/IRF and RPE atrophy.

**Fig. 1:**
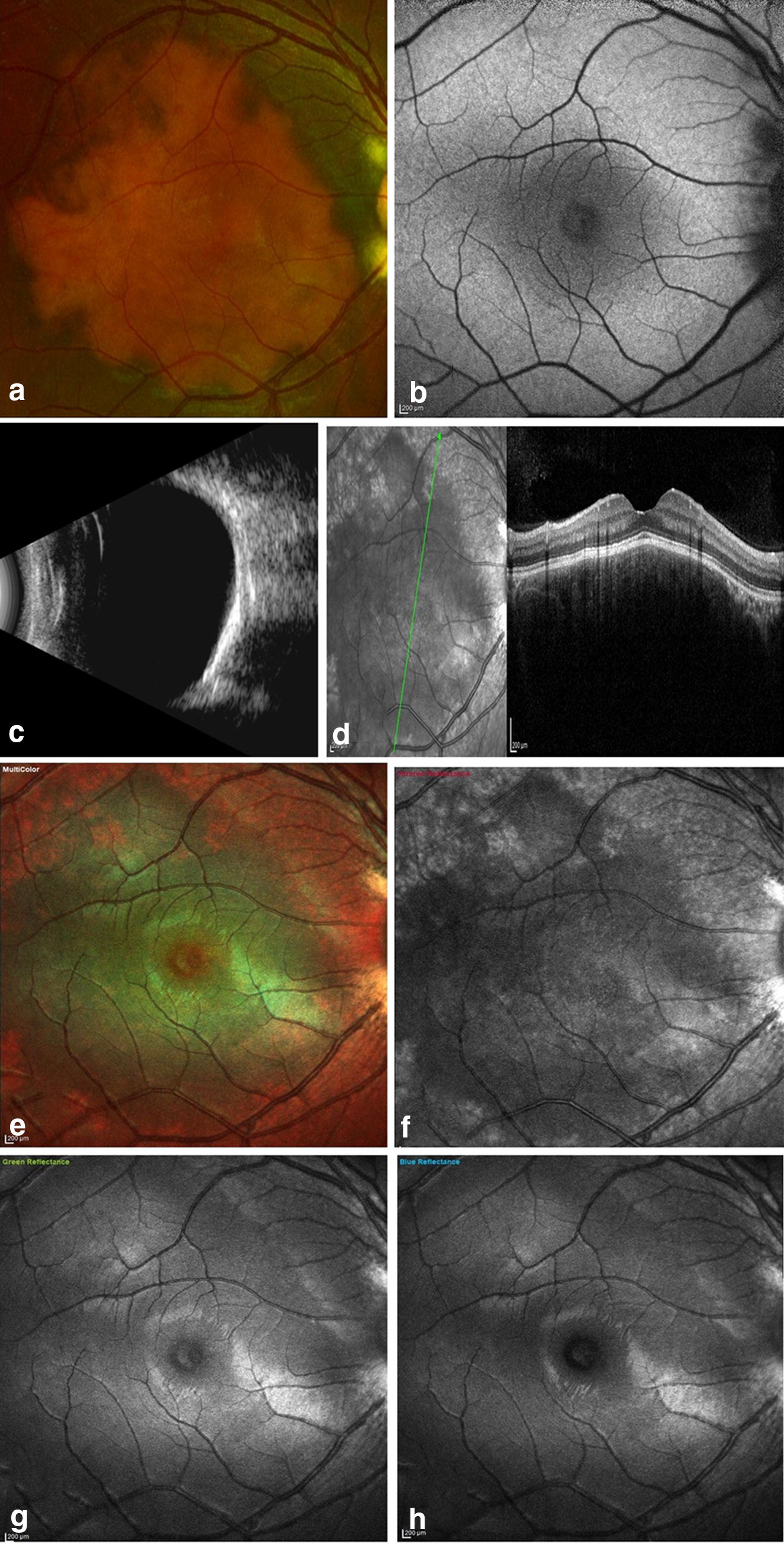
Case 1—Multimodal imaging with color fundus (CF), fundus autofluoroscence (FAF), optical coherence tomography (OCT), ultrasonography (USG) and multicolour (MC) imaging in a patient with RE calcified choroidal osteoma. **a** CF showing RE orange coloured lesion underneath the retinal pigment epithelium (RPE) at the posterior pole. **b** FAF showing iso autofluoroscence in the region of interest. **c** High bone density noted on USG b-scan. **d** Radial OCT scan passing thru’ the lesion shows retinal elevation with overlying normal retinal contour. **e** MC image showing the green colouration at the posterior pole. **f** Infrared reflectance showing dark areas corresponding to the tumour lesion. **g**, **h** Green and blue reflectance images do not show any changes due to CO at the posterior pole

**Fig. 2:**
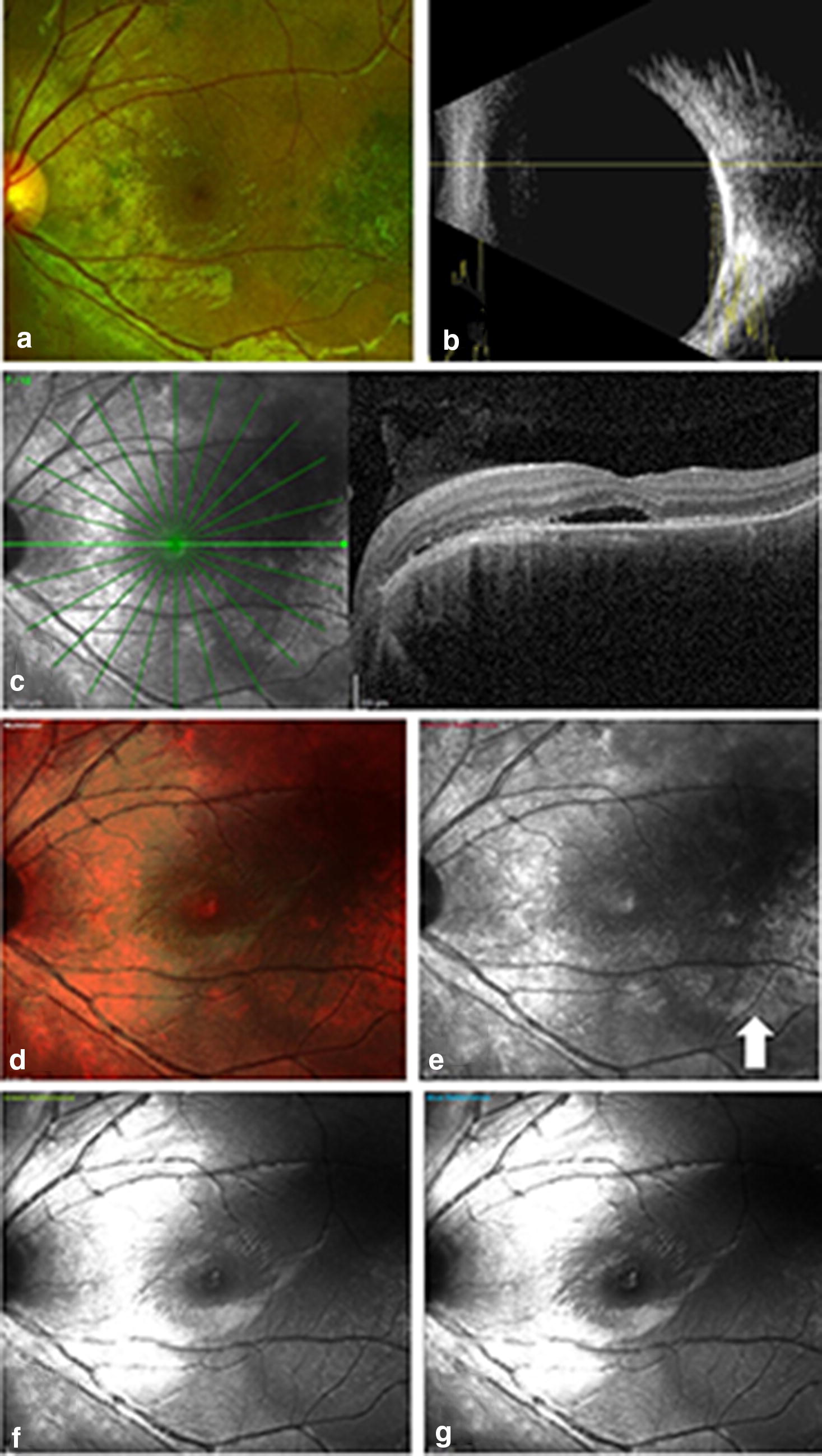
Case 3—Multimodal imaging in a patient with predominantly decalcified choroidal osteoma in the LE. **a** CF showing yellowish choroidal osteoma lesion at the peripapillary region with retinal pigment epithelial alterations noted temporal to the macula. **b** Ocular ultrasound showed hyperechoic area with shadowing confirming CO. **c** Horizontal line OCT scan passing through the fovea shows subtle elevation by the tumour temporal to the ONH and presence of sub foveal SRF. **d** MC image shows minimal orangish discoloration in the peripapillary area and temporal to the macula suggestive of overlying RPE atrophy. The decalcified tumour is not seen on MC image. **e** Infrared reflectance shows white areas corresponding to the orange areas in MC image suggestive of atrophied RPE. **f**, **g** Blue and green reflectance images do not show the osteoma lesion

**Fig. 3:**
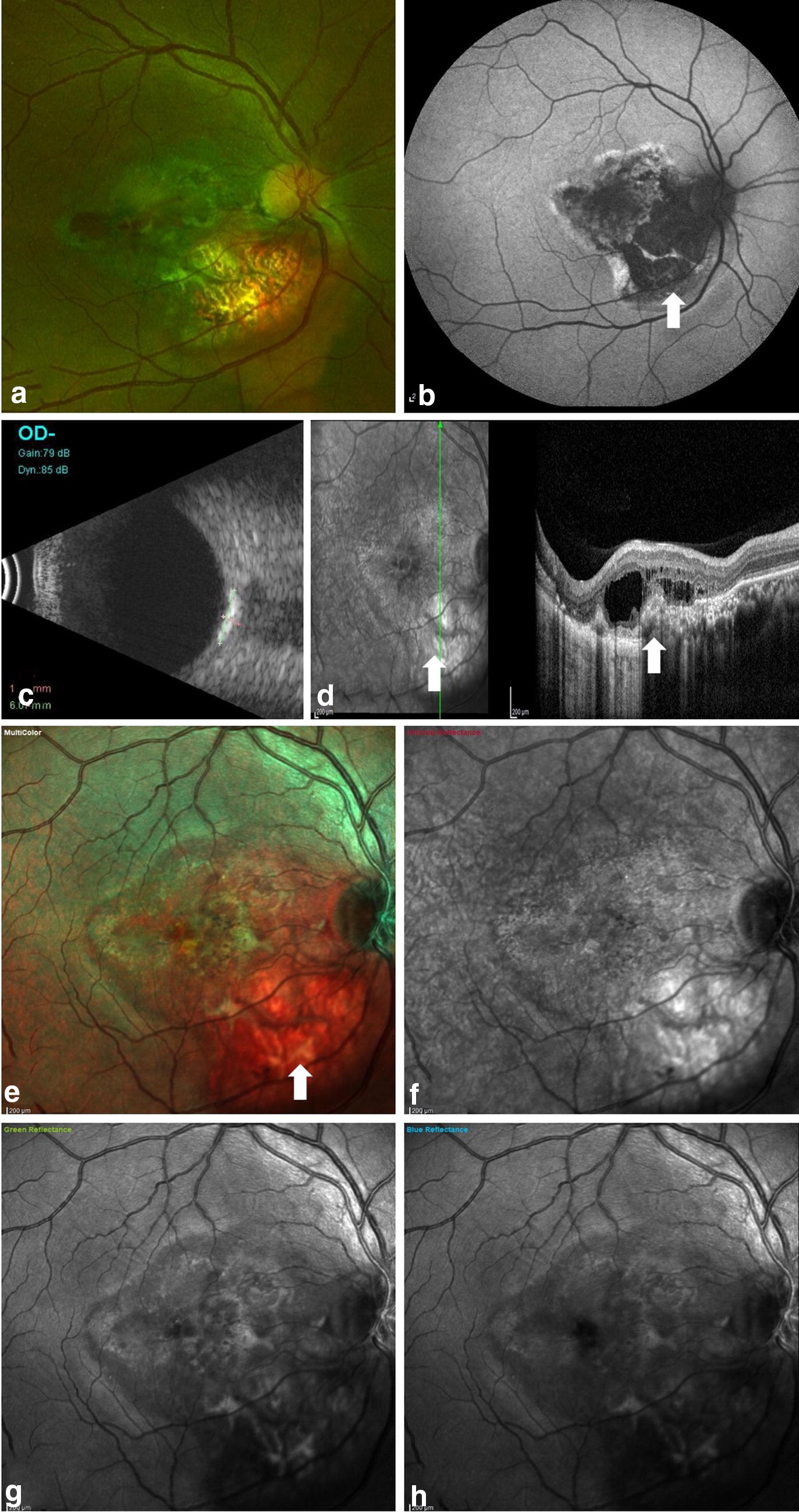
Multimodal imaging in case 5 with total decalcified choroidal osteoma. **a** CF showing a greyish white area inferotemporal to the optic nerve head (ONH) with RPE atrophy and prominent visible underlying choroidal vessels. **b** FAF showing areas of hypo AF suggestive of total RPE atrophy. **c** High bone density noted on USG b-scan. **d** Radial OCT scan passing vertically through the lesion shows disrupted RPE inferotemporal to the ONH with intraretinal fluid. **e** MC image shows orange discoloration at the posterior pole with bright orange area noted inferotemporal to the ONH suggestive of RPE atrophy overlying the choroidal osteoma. **f** Infrared reflectance shows white areas at the posterior pole and bright white area corresponding to the bright orange area noted on MC image suggestive of RPE atrophy. **g**, **h** Minimally white areas are noted in both green and blue reflectance images

**Fig. 4:**
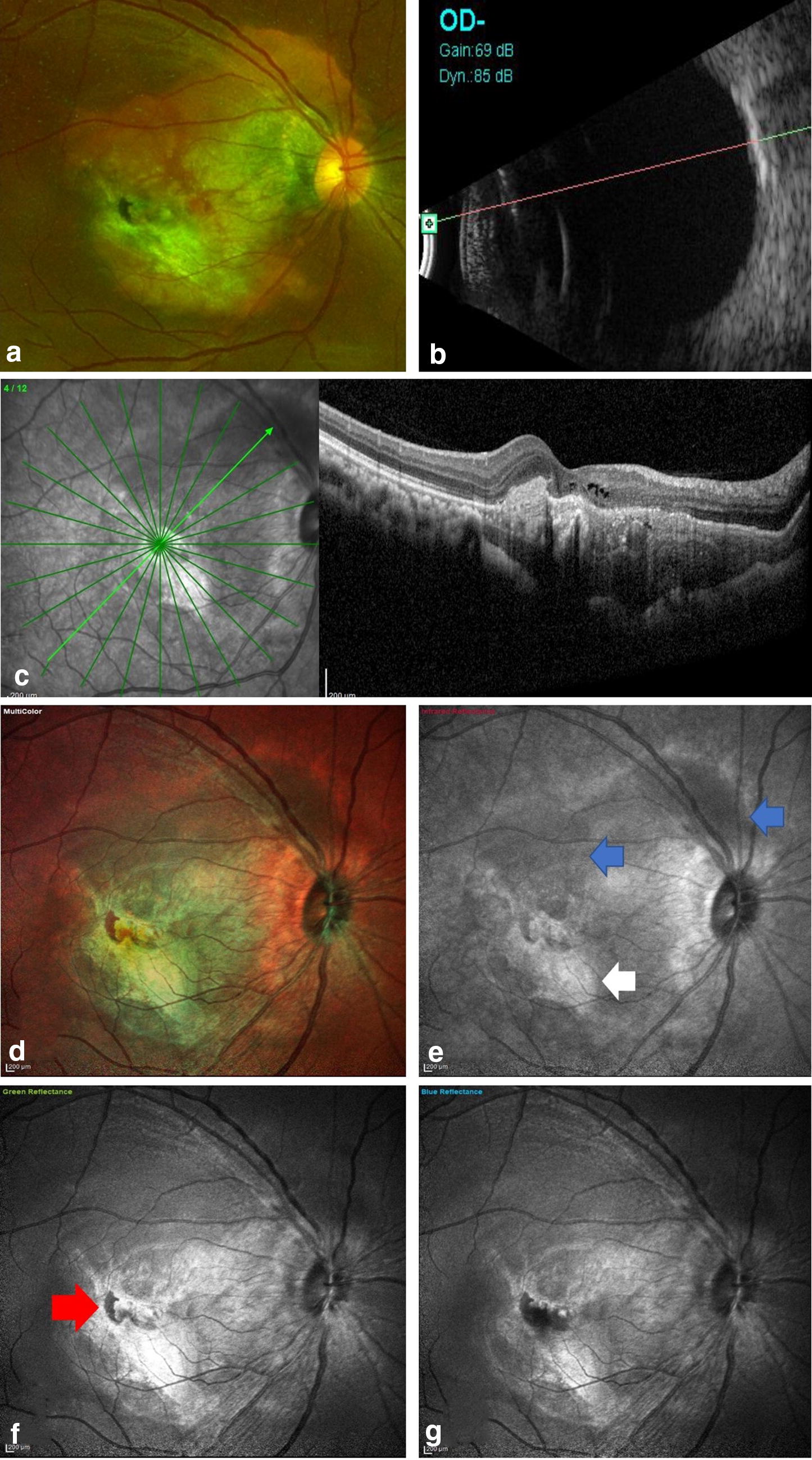
Multimodal imaging of a partly decalcified choroidal osteoma in the RE of case 6. **a** CF image of the RE showing CO lesion at the posterior pole with presence of CNV and subretinal hemorrhage. **b** High bone density noted on USG b-scan. **c** Horizontal radial OCT scan passing thru’ the macula demonstrating the presence of disrupted RPE with CNV and intraretinal fluid. **d** MC image shows green areas superior and temporal to the ONH suggestive of calcified CO. **e** Infrared reflectance showing dark areas corresponding to the calcifies areas of CO (blue arrows). Area of hyperreflectance is noted at the macula suggestive of CNV (white arrow). **g**, **h** Green and blue reflectance images shows bright white area corresponding to the CNV at the posterior pole while dark area is noted at the edge of CNV suggestive of hemorrhage (red arrow)

MC imaging was performed in all eyes using the cSLO Heidelberg Spectralis HRA-OCT (Heidelberg Engineering, Dossenheim, Germany) with a scanning field of 30° and sometimes 55°. IR images were the most efficient in identifying CO. The IR images identified ossified portions of the tumour as dark (hypo reflectance) areas while deossified portions showed iso-reflectance (no change) areas. White (hyper reflectance) areas were seen in RPE atrophy. The GR and BR images showed either no change or white areas depending upon the presence of hemorrhage and/or fluid in the retinal layers. On MC images, the color of the lesion varied from being green to no change to bright orange depending upon the tumour content and RPE atrophy. Green color was noted in calcified tumour while decalcified tumour showed no change on multicolour images. Orange areas were noted in eyes with areas of decalcified tumour with overlying RPE atrophy (Figs. 1, 2, 3, 4).

Seven eyes with SRF/IRF and clinical suspicion of choroidal CNV were imaged further with FA and ICGA and OCT- angiography in some cases. CNV was identified in 5 eyes and treatment were administered with anti-VEGF therapy in 4 eyes. One eye had scarred CNV; hence, it was observed.

## Discussion

Multi color scanning laser imaging is a recently introduced non-invasive innovative technology developed for Spectralis SD-OCT (Heidelberg Engineering, Heidelberg, Germany). In scanning laser ophthalmoscopy, a single point of laser light of a specified wavelength is scanned across the retina in a series of parallel lines and enabling an alternative method of capturing fundus images [8, 9]. It uses three laser colours, blue (488 nm), green (515 nm) and infrared (820 nm) that penetrate the tissue to different depths and simultaneously captures the reflectance strengths from different retinal structures and represents the information as en-face images. The infrared reflectance (IR) image visualizes structures at the level of the outer retina, RPE and choroid. Thus, the higher wavelength infrared light is useful in depicting and characterizing the CO lesion. The GR image allows imaging of retinal blood vessels, haemorrhages and exudates. The BR particularly provides details of the inner retina and the vitreoretinal interface such as epiretinal membranes, retinal nerve fibre layer (RNFL) thinning and macular pigment changes. Thus, secondary retinal findings like presence of intraretinal/subretinal hemorrhage, fluid or pigments are picked up with GR and BR images. The information from these 3 images are integrated to form a composite MC image [7]. In this study, we describe the features of CO on MC imaging and corelate them with that of CFP, FAF and OCT.

CO is a rare benign ossifying tumour of the choroid characterised by the presence of mature, spongy cancellous bone. On histopathology, dense bony trabeculae with marrow spaces traversed by pathognomonic dilated thin-walled blood vessels, termed spider or feeder vessels are seen. These vessels connect the choriocapillaris to the underlying larger choroidal vessels [1]. Choroidal imaging using the EDI-OCT has revealed the presence of bone lamella, tubular lamella with optically empty centre, vascular channels and trabecular bone in patients with CO [4, 5]. Because of its deeper location, CO are best picked up on IR images. The IR image shows hypo reflectance (dark) areas in an ossified tumour. This is probably because the 820 nm infrared light gets absorbed by the spongy bony trabeculae present in CO. As a result, very less quantity of light gets reflected back and hence, the lesion appears as a dark area. In decalcifying tumours, there is absence of spongy, bony trabeculae resulting in an iso-reflectance pattern of the tumour. Areas of RPE atrophy are identified as hyper reflectance (white) areas on IR image. In the BR and GR images, the CO lesion is not visible. This is due to the inability of the shorter blue and green wavelength light to reach the CO lesion. However, in the presence of secondary changes like pigmentary deposition and presence of intraretinal fluid and hemorrhage due to CNV, areas of abnormal reflectance are identified on BR and GR images.

Fundus autofluoroscence images does not show the osteoma lesion when the overlying RPE is intact. However, in areas of atrophic RPE, hypo AF (dark) areas are seen.

The MC image in CO shows color variations due to the variable reflectance produced by the tumour. The green colour on MC images are seen in eyes with calcified tumour while decalcified choroidal osteoma shows no colour change. Areas of RPE atrophy are seen as bright-orange areas. Pigment clumping over the tumour surface is usually difficult to identify ophthalmoscopically against the orange intrinsic tumour color. On MC images, pigment clumps are identified as yellow or orange spots while on IR images, they are seen as bright white spots.

Poor visual acuity in eyes with CO is usually seen when the decalcification with overlying atrophy of RPE and photoreceptors is located underneath the fovea. Thus, presence of bright orange areas on MC image and white areas on IR image at the sub foveal region can predict poor visual acuity. Hence, MC imaging can be considered as a useful tool in disease prognostication.

MC imaging has some advantages over CFP [10]. It is: (1) less photophobic to the patient; (2) can be used in undilated pupil; (3) 55° images gives a larger view of the periphery (4) can be combined with SD-OCT in a single device to allow simultaneous fundus and cross-sectional imaging and (5) provides images with high contrast and enables imaging through hazy media like cataract. MC imaging also seems to be superior to FAF in identifying the tumour and secondary changes produced by it. In comparison to FA and ICGA, MC imaging is non-invasive, less time consuming, cheaper, and has no side effects due to dye injection.

Our study has the advantage of having sufficient number of eyes with MC images in a rare disease like CO. We also had the multicolour image findings of the disease in its different stages; thus, helping us to understand better the role of MC imaging in CO. Our study had several limitations as well. Absent follow-up images meant inability to describe MC imaging changes over time. All ancillary imaging studies were not used in each patient, and raster scans on SD-OCT may have missed pertinent portions of the lesions. Larger series with longer follow-ups and comparison with other imaging modalities will elucidate greater utility of MC imaging for choroidal osteomas. Present study illustrates multicolour imaging features of CO lesion, which is hitherto not described in English language published literature.

## Conclusion

MC imaging is a useful tool in our arsenal of existing imaging modalities in the assessment of CO and its secondary changes. Changes in IR reflectance and MC images can be used as an indicator to assess the extent of tumour decalcification and its secondary changes and therefore, can aid in prognostication in the same. It has the potential to replace CFP in documentation and follow up of patients with CO.

## Abbreviations

CO: choroidal osteoma
US: ultrasound
FA: fluorescein angiography
OCT: optical coherence tomography
OCTA: optical coherence tomography angiography
EDI: enhanced depth imaging
MC: multicolour
CFP: colour fundus photography
FAF: fundus autofluoroscence
RPE: retinal pigment epithelium
SRF: subretinal fluid
IRF: intra retinal fluid
CNV: choroidal neovascular membrane
IR: infrared reflectance
GR: green reflectance
BR: blue reflectance
VEGF: vascular endothelial growth factor
SRH: subretinal hemorrhage
